# The development of drug resistance mutations K103N Y181C and G190A in long term Nevirapine-containing antiviral therapy

**DOI:** 10.1186/1742-6405-11-36

**Published:** 2014-11-21

**Authors:** Yuncong Wang, Hui Xing, Lingjie Liao, Zhe Wang, Bin Su, Quanbi Zhao, Yi Feng, Pengfei Ma, Jia Liu, Jianjun Wu, Yuhua Ruan, Yiming Shao

**Affiliations:** Beijing Center for Diseases Prevention and Control, Beijing, China; State Key Laboratory for Infectious Disease Prevention and Control, National Center for AIDS/STD Control and Prevention, Chinese Center for Disease Control and Prevention, Collaborative Innovation Center for Diagnosis and Treatment of Infectious Diseases, Beijing, China; Henan Center for Disease Control and Prevention, Zhengzhou, China; Anhui Center for Disease Control and Prevention, Hefei, China

**Keywords:** K103N, Y181C, G190A Navirapine (NVP), Antiviral therapy, Cohort study

## Abstract

**Objective:**

We built a cohort study of HIV patients taking long-term first-line Antiretroviral Therapy in 2003. In this assay, we focused on the development of primary drug resistance mutations against Non-Nucleoside Reverse Transcriptase Inhibitor (NNRTI), K103N, Y181C and G190A.

**Method:**

The cohort study was built in Henan province, China. We used Single Genome Amplification (SGA) to analyze the frequency of K103N, Y181C and G190A in serial plasma samples of three individual patients. We also performed standard genotype HIV drug resistance assay in 204 patients of this cohort study to analyze the frequency of these mutations.

**Result:**

In the SGA sequences, the K103N decreased and vanished, while the frequency of Y181C and G190A increased in individual patient receiving long-term Antiretroviral Therapy (ART). In the sequences of standard genotype HIV drug resistance assay, the frequency of K103N, Y181C and G190A had the similar pattern with that in SGA sequences. Among these patients, the viral suppression were still sufficient after receiving ART for 72 months, and 78.6% (160/204) patients could have their CD4 count over than 200cells/ul.

**Conclusion:**

In some patients, first-line ART had the possibility to provide sufficient treatment effect for over than 72 months, but in long-term treatment, the dominant NNRTI drug resistance mutation K103N could reduced, while the proportion of variants with mutation Y181C or G190A may increased. This result was not similar with that in vitro study, which state that variant with K103N or Y181C had an equal viral fitness with wild type.

## Background

Antiretroviral therapy (ART) in China started in 2003 [1], at the end of 2011, over 350,000 HIV-infected patients were receiving ART [2]. Chinese national treatment guidelines for ART is: (1) CD4 cell count, 350/mm^3^ (increased from 200/mm^3^ in 2008); (2) total lymphocyte count, 1,200/mm^3^; or (3) World Health Organization (WHO) stage III or IV disease [3]. The first-line regimen included two kinds of Nucleoside Reverse Transcriptase Inhibitors (NRTI) and one kind of Non-Nucleoside Reverse Transcriptase Inhibitor (NNRTI) [4]. Because of very limited antiretroviral drug formulations and lack of adequate laboratory monitoring of ART patients, most patients who initiated ART during the early phase of the free ART program had to stay on the same regimen for a long period of time (maximum for 112 months in my research). For this fact, the research on the development of drug resistance mutations in long-term ART became more and more necessary.

NNRTIs is a crucial component of Chinese ART, it exhibit a longer plasma half-life and a longer duration of detectable levels than nucleoside analog reverse transcriptase inhibitors (NRTIs) [5]. Nevirapine (NVP) is an acceptable first-line NNRTI for HIV-1-infected patients [6] and is known as the most common NNRTI used in the ART regimens of China. In our research, all patients took NVP as NNRTI in their regimens, and were never been stopped and changed.

NVP is a potent NNRTI to fight against HIV, but it has two drawbacks: one, it could cause allergic reactions in approximately 5-14% of users worldwide [7–9], another one is that NVP has a low genetic barrier; a single mutation can lead to a high-level drug resistance against NVP [10, 11]. Among the NNRTI drug mutation, K103N, Y181C and G190A are the most common mutation associated with resistance to NVP [12, 13]. Even in the patients before ART, Low frequencies of K103N and Y181C are significantly associated with an increased risk of virologic failure [14]. In vitro study, K103N, Y181C and G190A could not provide significant fitness reduction to HIV viruses, variant with these mutations had similar viral replication fitness with the wild type variant [15, 16]. In the past, consider the low fitness reduction and high-level resistance of NNRTI associated mutations, researchers seldom paid attention to the NNRTIs resistant profiles resulting from therapy failure, because a single NNRTI treatment failure always means the second NNRTI therapy failure [17, 18], and in countries with rich ART resources, patients with NNRTI drug resistance mutations would switch to new regimen but in China, a country with limited antiretroviral drugs, patients had to took same NNRTI drug for long time, so the understanding of the development of NNRTI associated mutations became very necessary.

In this essay, we retrospectively studied the development of K103N, Y181C and G190A in long-term ART.

## Method

### Study population

National Center for AIDS/STD Control and Prevention built an observational cohort study enrolled 339 patients from Queshan County, Henan Province from December 2003 to December 2004. Patients who were 18 years of age or older and started ART between 2003 and 2004 were included. All patients agreed to participate in the study through informed consent. Patients were followed every 6 months up to 11 November 2012. Due to stopping ART, death or losing to follow-up. By the end of 2012, among these 339 patients, 75 patients died for AIDS associated diseases, 34 patients stopped ART, and 26 patients lost to follow-up, so we had 204 patients for this study.

204 patients in this cohort study were all treated with AZT/ddI/NVP (AZT, 300 mg 2 times/day; ddI, 200 mg 2 times/day; NVP, 200 mg, 2 times/day). Treatment adherence was assessed by questionnaire in each follow-up. The number of patients who had over 95% adherence in each follow-up showed no significant difference (p > 0.05).

Sample collection was approved and done by Henan CDC. In each follow-up, Neibor Joining phylogenetic analysis was performed to confirm that the longitudinal samples were from the same patient, and all the information of patients and the results of drug resistance assays were all well stored by China CDC. All patients took Nevirapine (NVP) as the NNRTI in regimen from the initial time and have never been changed (Table 1).

**Table 1:** **Characteristics of HIV-infected study population on antiretroviral cohort study Henan Province, central China**

Characteristics	N (%) N = 339
Sex	
Male	143 (42.2)
Female	194 (57.8)
Median age at inclusion	39 (25–64)
Education	
Primary school or less	218 (64.2)
Middle school or more	119 (35.8)
Marital status	
Married or living with partner	287 (84.6)
Single, divorced or widowed	52 (15.4)
Occupation	
Farmer	327 (96.5)
Other	12 (3.5)
Risk exposure	
Former plasma donor	327 (96.5)
Sexual transmitted	7 (2.1)
Unknown	5 (1.4)
Original combination antiretroviral regimen	
Zidovudine, didanosine, nevirapine	288 (85.0)
Stavudine, didanosine, nevirapine	51 (15.0)
Stopped cART	34 (10.0)
Patients replacing ddI to 3TC	117 (34.5)

To assess the ART outcome, the date of virologic failure was defined as the first recorded date of plasma viral load of more than 5000 copies/ml after 6 months of treatment, as the 2010 WHO guideline on antiretroviral therapy for HIV infection in adults and adolescents (WHO. Antiretroviral therapy for HIV infection in adults and adolescents: recommendations for a public health approach. 2010). Similarly, the date of immunologic failure was also defined by WHO criteria as the earliest date of any one of the following after 6 months of treatment: posttreatment CD4+ cell count falling to or below baseline CD4^+^ cell count; 50% decrease from peak CD4^+^ cell count; or two consecutive CD4^+^ cell counts of less than 100 cells/ml or last CD4^+^ cell count of less than 100 cells/ml. Baseline CD4^+^ cell count was defined as the last pretreatment count within 6 months of treatment. If no pretreatment CD4^+^ cell count was done, the earliest count within 1 month of starting treatment was used for the baseline value.

In this study, we used plasma specimens of four time points: treatment for 6–18 months; 30-42 months; 54-67 months and 75-102 months. Among the 204 patients, we select 3 patients for Single Genome Amplification (SGA). The criteria was (1) at least 6 time points; (2) no stopping and restarting ART, (3) the results of CD4 count and viral load were all available.

### Laboratory methods

Standard genotype HIV drug resistance assay was performed on the specimens in the four time points mentioned above. SGA were performed in 3 patients. They were all performed in the National center for HIV/STD diseases control and prevention, China CDC. In each follow-up, viral load assays were performed by M2000 Biosystem (Abbott Company, USA). The CD4 cell count for all patients were performed by FACSalibur flow cytometer (BD Company, USA).

### Viral RNA extraction and standard genotyping

Viral RNA were extracted from plasma samples using QIAamp Viral RNA Mini Kit (QIAGEN company). We used nested RT-PCR process to get a 1.3 kb (2253–3553) fragment of pol gene, the fragment includes Protease amino acid codons 1–99 and Reverse transcriptase amino acid codons 1–300. The sequences of primers for the first round RT-PCR process could be seen below: MAW265′-TTGGAAATGTGGAAAGGAAGGAC-3 and RT215′-CTGTATTTCTGCTATTAAGTCTTTTGATGGG-3′. PCR temperature was 50°C, 30 min for reverse transcription, 94°C, 10 min for preheat, followed by 94°C, 30 s; 55°C, 30 s; 72°C, 2 min 30 s, 30 cycles. 72°C 10 min for final amplification. In the second round, we got a 1.3 kb fragment using primer RT205′-CTGCCAGTTCTAGCTCTGCTTC-3′ and PRO15′-CAGAGCCAACAGCCCCACCA-3′. PCR temperature was 94°C,10 min for preheat, followed by 94°C, 30 s; 55°C, 30 s; 72°C, 2 min 30 s, 30 cycles. 72°C 10 min for final amplification.

### Single genome amplification (SGA)

RNA was extracted from plasma specimens by QIAamp Viral RNA Mini kit (Qiagen, Germany), and was reverse transcript to cDNA by SuperScriptIII protocol according to the manufacturer’s instructions (Invitrogen LifeTechnologies, USA). We then dilute cDNA to a moderate concentration in 96-well plates, such that fewer than 29 PCRs yielded an amplification product. According to probability theory, once less than 30% PCR reactions get positive results in 96-well plates, it has more than 80% to get each sequence amplified from a single genome [19, 20].

We used 3730 Sequencer (ABI company, USA) to get sequences from all the positive amplification products, and use Sequencher (4.10.1) to clean and edit sequence data. In an individual sequence, if there is no ambiguous base pair, it was confirmed that this sequence amplified from a single genome. Finally, we got about 20 such single genome sequences for each time point.

### Data analysis (Ka/Ks Ratio of NNRTI associated mutations)

Ka/Ks ratio is a tool to characterize selection pressure of observed amino acid mutations over observed synonymous mutations, the ratio of the number of non-synonymous substitutions per non-synonymous site (Ka) to the number of synonymous substitutions per synonymous site (Ks) [21]. Ka/Ks ratio was used as an indicator of selective pressure acting on a protein-coding gene. A Ka/Ks value of 1 indicates neutral selection, which means amino acid changes are neither being selected for nor against. A Ka/Ks value of < 1 means negative selection pressure. That is, most amino acid changes are baneful and are selected against. The positive selection (Ka/Ks > 1) is much rare, indicating that amino acid changes are favored and may be useful [22].

The concept of correlated mutations is one of the basic ideas in evolutionary biology. The amino acid substitution rates are expected to be limited by functional constraints. Given the functional constraints operating on gene, a mutation in one position can be compensated by an additional mutation. Then mutation patterns can be formed by correlated mutations responsible for specific conditions. Here, we used conditional Ka/Ks ratio (cKa/Ks), which is a metric to measure the effects of mutations at one site on the selection pressure for mutations at another site.

We conducted cKa/Ks ratio analysis on two dataset, one is RT gene sequences of 204 patients using standard HIV genotype sequencing, the other one is RT gene sequences of three patients using Single Genome Amplification. In the two dataset, we had two time points, one was receiving ART for 12 months (10–14 months), and the other time point was receiving ART for over 60 months (56–73 months).

In dataset one, because it is nearly impossible to get gene sequence from plasma samples with suppressed viral load, there were 75 sequences for the first time point, and 74 for the second time point, in dataset two, we had 99 sequences for the first time point and 109 for the second time point. All the cKa/Ks analysis was done by R Language, using the package CorMut (1.2.0, Zhenpeng Li ).

### Ethics approval

Chinese Center for Disease Control and Prevention research ethics boards approved this study.

## Result

### General information

Because of transferring to another clinic center, immigrating to other town, not all the 204 patients participated in four follow-ups of this cohort study. There were 184, 148, 200 and 182 patients participated in each follow-up respectively.

The median treatment duration [M(P25 ~ P75)] of the 204 patients was 86.4 months (77.6-98.0 mo). There were 46 patients receiving ART for over 100 months (22.5%). Among the 204 patients, the main age range was 30–45 years (56.4%), maximum for 63, minimum for 24. Male female ratio was 0.6:1.

### The frequency of K103N, Y181C and G190A in SGA

The frequencies of three primary NNRTI drug resistance mutations, K103N, Y181C and G190A, fluctuated during long-term ART. More specifically, K103N decreased, while Y181C and G190A showed increasing trend (Table 2).

**Table 2:** **NNRTI mutations and treatment information for three individual patients in SGA**

ART duration	Patient ID	Viral load	CD4 count	K103N	G190A	Y181C	K101E	SGA quantity
20.5	HENDRC593	500	498	6 (35.3)	11 (64.7)	6 (35.3)	11 (64.7)	17
27.5		500	691	8 (42.1)	11 (57.9)	8 (42.1)	11 (57.9)	19
38.9		2976	770	4 (26.7)	11 (73.3)	7 (46.7)	11 (73.3)	15
44.8		91000	655	3 (15.8)	16 (84.2)	5 (26.3)	16 (84.2)	19
49.9		120000	595	0 (0.0)	23 (100.0)	2 (8.7)	23 (100.0)	23
61.5		52000	696	0 (0.0)	16 (94.1)	5 (29.4)	16 (94.1)	17
76.1		170000	551	0 (0.0)	20 (100.0)	9 (45.0)	18 (90.0)	20
23.9	ANHDRC0106	44730	222	10 (100.0)	1 (10.0)	10 (100.0)	0 (0.0)	10
30.1		159000	196	5 (41.7)	3 (25.0)	12 (100.0)	7 (58.3)	12
35.5		94700	255	5 (33.3)	3 (20.0)	15 (100.0)	10 (66.7)	15
42.6		379000	153	3 (21.4)	3 (21.4)	14 (100.0)	11 (78.6)	14
46.4		17600	294	0 (0.0)	11 (84.6)	13 (100.0)	13 (100.0)	13
52.6		33100	256	1 (7.1)	10 (71.4)	14 (100.0)	13 (92.9)	14
20.5	HENDRC622	2076000	104	10 (83.3)	10 (83.3)	0 (0.0)	2 (16.7)	12
27.4		7263	164	14 (93.3)	15 (100.0)	0 (0.0)	1 (6.7)	15
33.0		17160	142	12 (75.0)	16 (100.0)	0 (0.0)	4 (25.0)	16
44.7		13160	138	9 (81.8)	11 (100.0)	0 (0.0)	2 (18.2)	11
49.9		66000	176	9 (90.0)	10 (100.0)	1 (10.0)	1 (10.0)	10
56.8		81000	130	4 (22.2)	18 (100.0)	2 (11.1)	5 (27.8)	18
61.5		39000	181	8 (42.1)	19 (100.0)	6 (31.6)	1 (5.3)	19

In the patient HENDRC593, the frequency of K103N showed a decreasing trend, especially from 44.8 months, the K103N vanished in SGA sequences. On the contrary, G190A showed a dramatic increasing trend from the initiation of ART.

In patients ANHDRC106, after taking ART for 23.9 months, K103N and Y181C became dominant mutations (100%), while the frequency of G190A was 10%. In the follow-up time points, K103N showed a decreasing trend and G190A showed an increasing trend, while Y181C maintained dominant.

In patients HENDRC622, K103N showed a decreasing trend from 20.5 to 61.5 months, while G190A kept 100% in quasispecies. The Y181C increased slightly from 0.0% to 31.6% from receiving ART for 44.7 months to 61.5 months

The research of Jiong Wang [23] stated that mutation K101E + G190S replicate better with NNRTI drug. Here we showed the frequency of K101E in each time points. It is interesting that the frequency of K101E changed with G190A. In patient HENDRC593, the frequency of K101E increased from 64.7% to 100% in 49.9 months, while the frequency of G190A increased exactly from 64.7% to 100% in 49.9 months. In patient ANHDRC106, the frequency of K101E increased from 0% to 100% in 46.4 months, while the frequency of G190A increased from 10% to maximum 84.6% in 46.4 months. In patients HENDRC622, the frequency of G190A kept in 100% in nearly all time points, while the frequency of K101E kept under 30%.

### Standard genotyping: the frequency of K103N, Y181C and G190A in long-term cohort study

**Figure 1:**
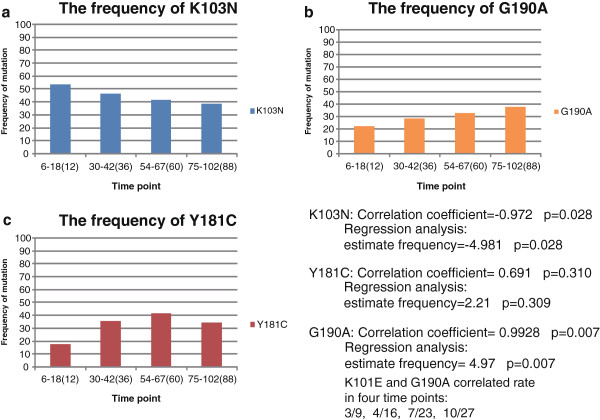
**The frequencies of three NNRTI drug resistance mutations in long-term ART treatment. a**. the frequency of K103N with treatment duration; **b**. the frequency of G190A with treatment duration; **c**. the frequency of Y181C with treatment duration.

All drug resistance assays were done by 20 December 2012. All the 204 patients took NVP from the initiation of ART treatment and never changed to another NNRTI drug. We collected the treatment and drug resistance results in 4 time points of the 204 patients: 1.ART for 6-18 months; 2. ART for 30-42 months; 3. ART for 54-67 months; 4. ART for 72-102 months. Then we calculated and analyzed the frequency of K103N, Y181C and G190A in the four time points (Figure 1). Frequency of K103N showed a decreasing trend while both the frequency of Y181C and G190A showed an increasing trend. Specifically, the frequency of K103N had a negative correlation with treatment duration, and mutation G190A had positive correlation with treatment duration. The frequency of mutation Y181C and treatment duration did not show significant correlation.

We also analyzed the frequency of K101E in 204 patients, but here we showed the correlated rate of K101E and G190A in four time points, they were 3/9, 4/16, 7/23, 10/27 (about 30%).

### Treatment effect in long-term Nevirapine-contain ART

At the time point of receiving ART for 6–18 months, 84.8% patients had their CD4 cells counts over 200 cells/ml. At the time point of receiving ART for over 72 months, there were still 78.6% (143/182) patients had their CD4 counts over 200 cells/ml.

For viral load, there were 39.1% patients failed to suppress their viral load in initial period (6–18 months). After receiving ART for over 72 months, 44.5% patients failed to suppress their viral load; it indicated that the first-line ART had the possibility to provide treatment effect in 55.5% patients (Table 3).

**Table 3:** **Treatment effect of long-term ART in 204 patients**

Treatment duration(months)	Number of patients	Patients with CD4 > 200 cells/μl	Patients with VL > LDL	Patients with NNRTIdr and CD4 > 200 cells/μl	Patients with NNRTIdr
6-18	184	156 (84.8%)	72 (39.1%)	34 (77.3%)	44
30-42	148	114 (77.0%)	95 (64.2%)	43 (76.8%)	56
54-67	200	147 (73.5%)	105 (52.5%)	49 (70.0%)	70
72-102	182	143 (78.6%)	81 (44.5%)	56 (77.8%)	72

notes:1. The number in this table refer to the number of certain patients, while proportions are in brackets.

2. VL refer to viral load,LDL refer to the lowest detectable viral load of certain method.

Besides the 204 patients, we had three patients performed SGA experiments, all of them had received ART for over 50 months, the maximum one is 76.1 months. After long-term ART, the viral load were failed to be suppressed sufficiently, but the CD4 count were kept steady and were similar with the initial time of ART. The long-term ART appeared to postpone the development of HIV virus.

### The correlated mutations with K103N, Y181C and G190A (result of cKa/Ks ratio)

In the result of cKa/Ks analysis all positively correlated mutations (cKa/Ks > 1) were listed here in Tables 4 and 5, mutations with cKa/Ks smaller than 1 were not included in the tables. Mutations in Position 1 were the condition mutations, mutations in Position 2 were the accessory mutations that were favored by ART with mutations in Position 1. It showed that M41L, D67N, T69D, K70R, and K219R were the most common NRTI mutations that associated with the three NNRTI mutations, K103N, Y181C and G190A. In NNRTI mutations besides K103N, Y181C and G190A themselves, H221Y was the most common NNRTI mutations associated with the three NNRTI mutations.

**Table 4:** **Correlated mutations in standard genotype sequencing dataset**

	Position1	Mutation type	Position2	Ckaks	Lod
ART for 12 months	K103N	NRTI	M41L	1.5	7.05
		NNRTI	Y181C	1.29	5.17
			G190A	1.6	5.22
			H221Y	5	2.87
	Y181C	NRTI	None	None	None
		NNRTI	H221Y	6	3.45
	G190A	NRTI	None	None	None
		NNRTI	K103S	3	Inf
ART for over 60 months	K103N	NRTI	T69N	1.33	2.39
			K70R	1.17	3.32
		NNRTI	Y181C	1.27	7.16
			H221Y	2.75	5.5
	Y181C	NRTI	D67N	1.8	3.77
			T69N	2.5	5.85
			K70R	5	4.95
		NNRTI	K103N	2	15.78
			H221Y	4	6.05
	G190A	NRTI	D67N	4.8	3.52
			T69N	4	5.71
			K70R	3	3.52
		NNRTI	K101E	6	3.52
			Y181C	1.5	4.11
			K103S	10	Inf

**Table 5:** **Correlated mutations in Single Genome Amplification dataset**

	Position1	Mutation type	Position2	Ckaks	Lod
ART for 12 months					
	K103N	NRTI	D67N	5	2.93
			T69D	8	Inf
			K70R	16	9.37
		NNRTI	Y181C	26	15.23
			H221Y	17	9.96
	Y181C	NRTI	D67N	5	2.93
			T69D	8	Inf
			K70R	1.12	5.27
		NNRTI	K103N	2	29.44
			H221Y	17	9.96
	G190A	NRTI	None		
		NNRTI	K101E	29	16.98
ART for over 60 months	K103N	NRTI	T69D	12	Inf
		NNRTI	K219R	88	6.75
	Y181C	NRTI	D67N	11.5	14.12
			T69D	2	Inf
		NNRTI	K103N	2.25	9.41
			H221Y	26	15.96
	G190A	NRTI	M41L	21.25	88.84
			D67N	7.33	13.51
			T69D	12	Inf
		NNRTI	K101E	13.8	42.37
			K103N	12	11.47
			Y181C	6.67	24.56
			H221Y	3.33	12.28

Note: **Ckaks** indicates the ka/ks ratio of two mutations. Here, it stands for the ka/ks ratio for M184V and the correlated mutations.

**Lod** is the confident score for positive selection, If LOD >2, the positive selection is significant.

**Inf** indicates the maximum infinity.

Among K103N, Y181C and G190A, some correlations could be found, but they were not always positively correlated, the correlated mutations network changed with the time of ART. For example, in standard genotype sequencing dataset when receiving ART for 12 months Y181C, G190A and H221Y were positively correlated with K103N, but when receiving ART for over 60 months, Y181C and H221Y were positively correlated with K103N, while G190A was not.

Besides the mutations conferring drug resistance effect, there were a number of accessory mutations or compensatory mutations that correlated with the three NNRTI mutations, more specifically, 5 mutations for G190A in 12 months 14 ones for K103N, and 9 for Y181C. In the dataset of receiving ART for over60 months, there were 35 mutations that correlated with G190A, 20 mutations for K103N, and 26 mutations for Y181C. It indicated that with the ART extended, more accessory mutations would emerged.

## Discussion

This research was based on a cohort study including 204 patients taking first-line ART therapy since early stage infection. By the end of 2012, the median treatment duration was 86.4 months (77.6-98.0 months), including 46 patients received ART for over 100 months (22.5%). In our research, after the ART treatment for over 72 months, 55.5% patients had their viral load suppressed and 78.6% patients kept CD4 counts over 200 cells/ml. It seems that the first-line ART could still possibly be effective after long-term treatment.

Though the first-line ART is potent method to fight against HIV, but the development of drug resistance could make ART less effective [24–27]. NNRTI associated drug resistance mutations could compromise the success of NNRTI-containing ART [28–30]. Among these mutations, K103N Y181C and G190A are the most frequent NNRTI associated drug resistance mutations. In a research on nevirapine-experienced individuals, the frequencies of these mutations were 43%, 46% and 26% respectively [31]. Among the three NNRTI associated mutations, K103N is one of the most clinically important NNRTI resistance mutations; it causes 20- to 50-fold resistance to most available NNRTIs [32, 33]. In phenotypic experiments, K103N, Y181C and G190A were defined as the mutations that have almost the same replication fitness with wild type. The rank of fitness among mutant viruses was as follows: wild type (WT) ≥ Y181C ≥ K103N ≥ G190A ≥ V106A ≥ P236L ≥ G190S [34, 35]. Besides the replication fitness, K103N can persist for a long time in naive patients, over 2 years in a present case report [36]. K103N also can be found in blood mononuclear cell (PBMC) DNA, with no K103N observed in plasma [37, 38]. All the previous research indicated that K103N can steadily exist in plasma and PBMC, and quasispecies with K103N could be more possibly to survive in the surrounding with NVP.

In our research, the result was different with that in vitro study. We found that during the long-term ART treatment, the frequency of K103N, Y181C and G190A were not kept steady, the fact is the frequency of quasispecies with K103N decreased, and could even vanish in SGA sequences, while the proportion of Y181C and G190A could increase from 0% to nearly 100%. This result was also identified in a 204-patinets long-term cohort study.

Based on the results, we estimated that among the three strong NNRTI drug resistance mutations, K103N, Y181C and G190A, there may be some competition process in long-term ART. In the initial period of NVP-containing ART, K103N, Y181C and G190A emerged in different rate among quasispecies which K103N and G190A had a higher rate than Y181C [39]. Consider the fact that three mutations have similar replication fitness and the replication fitness which are associated with mutant prevalence in vivo [40–42]. Mutation emerged late would develop to the proper proportion associated with its replication fitness, while the proportion of mutation emerged earlier would reduced because the late emerged mutation took part of its dominant role. Then these three mutations would finally break the imbalanced and unequal proportion which was established in initial ART period.

The result of cKa/Ks ratio could provide another support for the competition hypothesis. Among the K103N, Y181C and G190A, they were not always positively correlated with each other, but may turn to negative correlation with the time of receiving ART. It indicated that although in vitro study, the three mutations all had high replication rate and less viral fitness reduction than other NNRTI mutations, variants with these mutations were not so strong in vivo when existed in plasma at same time, they were not always favored by variant under ART pressure. Some competition effect among them may change the frequency of these mutations.

Here is a question according to the research of Jiong Wang [42], the reduced fitness for G190A mutants may not explain the increased frequency of G190A variants over time. It is true that G190A alone can reduce viral fitness, but another research of Jiong Wang [23] found that the variant with K101E + G190S replicated better in the presence of NNRTIs than in the absence of drug, it indicate that mutation K101E may have some effect on the mutant of Codon 190 to enhance the viral fitness. In our study, the frequency of K101E had a similar trend with that of G190A in SGA result, and nearly 30% (3/9, 4/16, 7/23, 10/27) K101E emerged with G190A in standard genotyping result. This may partly explain the question that the increasing of G190A frequency in long-term ART.

The further mechanism and detail of the competition process were remaining unclear; we may need next-generation sequencing and other techniques to go deep in this question. At least we believe this changing of NNRTI mutations is crucial in long-term ART treatment, especially for the country with limited antiviral drugs.

## Abbreviations

HIV: Human immunodeficiency virus
ART: Antiretroviral therapy
NRTI: Nucleoside reverse transcriptase inhibitors
NNRTI: Non-nucleoside reverse transcriptase inhibitors
PCR: Polymerase chain reaction
TAMs: Thymidine analogue mutations.
